# Bilateral Triple Concha Bullosa: A Very Rare Anatomical Variation of Intranasal Turbinates

**DOI:** 10.1155/2014/851508

**Published:** 2014-11-30

**Authors:** Turhan San, Selma San, Emre Gürkan, Barış Erdoğan

**Affiliations:** ^1^ENT Department, Medeniyet University Göztepe Education and Research Hospital, Istanbul, Turkey; ^2^Anesthesia and Reanimation Department, Haseki Education and Research Hospital, Istanbul, Turkey; ^3^ENT Department, Haydarpaşa Education and Research Hospital, Istanbul, Turkey; ^4^ENT Department, Bolvadin State Hospital, Afyonkarahisar, Turkey

## Abstract

Pneumatization of the intranasal turbinates or concha bullosa is an anatomic variation of the lateral nasal wall. Concha bullosa is defined as the presence of air cells in turbinates. It can be best diagnosed with paranasal sinus computed tomography. Concha bullosa is a possible etiologic factor for recurrent sinusitis due to its negative effect on paranasal sinus ventilation and mucociliary clearance. Concha bullosa is most commonly seen in the middle turbinate and less frequently in the inferior or superior turbinate. Pneumatization of all turbinates is very rare. To our knowledge, there are only two publications about a case with concha bullosa in all turbinates in the current literature. Here, we present a woman with bilateral pneumatization in all three intranasal turbinates.

## 1. Introduction

Intranasal turbinates are required structures for the maintenance of normal nasal functions such as humidification, hydration, lubrication of the upper respiratory system, olfaction, filtration, and thermoregulation. The reason of pneumatization of the intranasal turbinates is still unknown. Although mostly asymptomatic, overpneumatized turbinates may constitute mass effect and in these cases as a result of impaired ventilation and drainage of osteomeatal region it can lead to sinusitis.

## 2. Case Presentation

A 20-year-old woman was admitted to our clinic with nasal obstruction, postnasal drip, and intermittent headache for 3 years.

Anterior rhinoscopy and nasal endoscopic evaluation revealed that the nasal septum was deviated to the left, bilaterally inferior and middle conchae were hypertrophied, and the nasal mucosa was normal.

**Figure 1:**
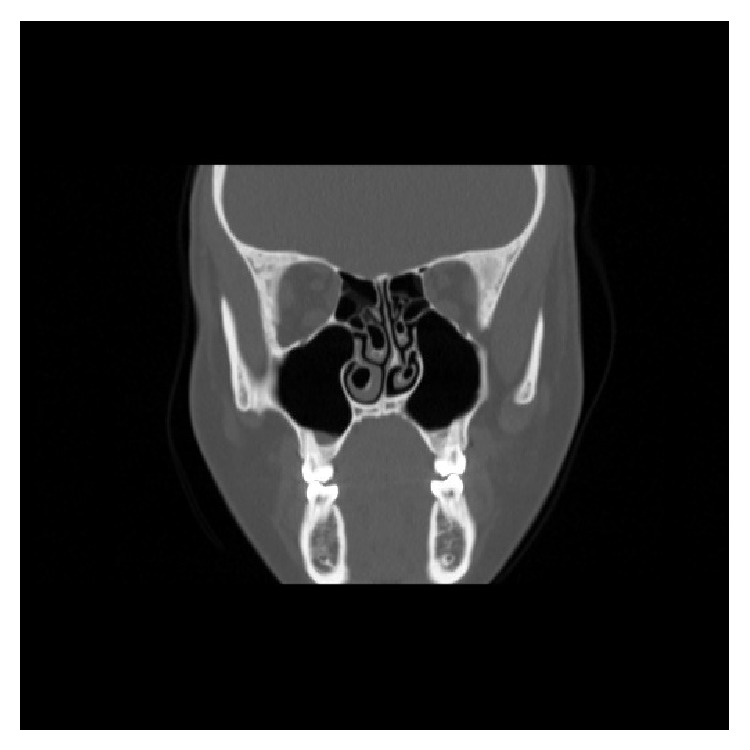
Coronal computed tomography section showing bilateral middle and inferior turbinate pneumatization and nasal septal deviation to left.

**Figure 2:**
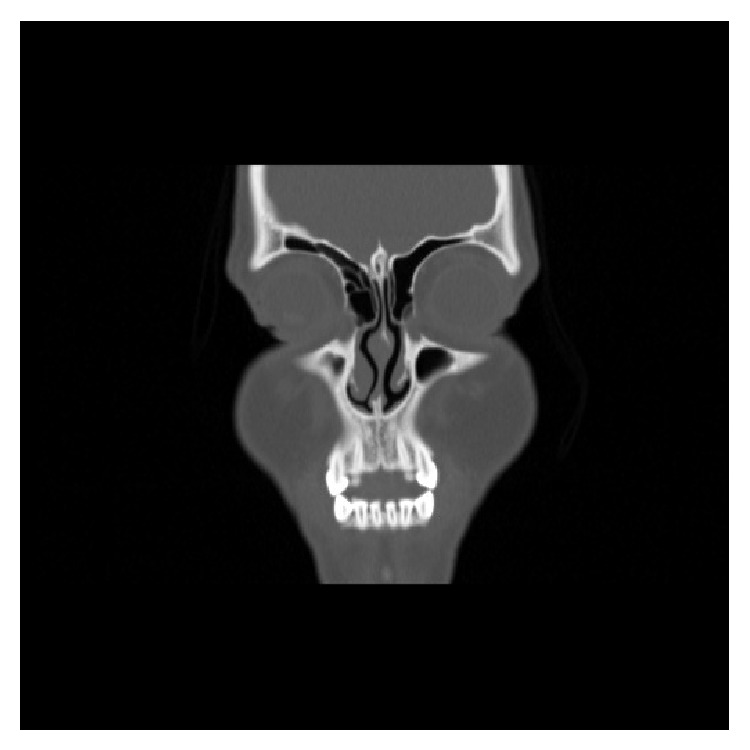
Coronal computed tomography section showing bilateral superior pneumatization.

Paranasal sinus computed tomography (CT) scan with coronal plane views revealed that the inferior, middle, and superior turbinates were pneumatised bilaterally, a moderate nasal septum deviation to the left with a spur formation and inflammatory mucosal thickening in the right maxillary sinus (Figures 1 and 2).

After giving information to the patient about the surgical procedure we performed an endoscopic surgery including lateral marsupialisation for both middle turbinate pneumatizations, a limited septoplasty and crushing and outfracture of the inferior turbinates. For both inferior turbinate pneumatizations, the bullous structures were reduced by crushing and outfracture. Superior turbinates were left intact since they had no visible clinical significance. She was discharged from the hospital 1 day after the operation and any postoperative complications were not seen. Follow-up evaluation indicated that the patient's symptoms had improved dramatically and she had no further complaints.

## 3. Discussion

Concha bullosa (CB) is the most common anatomical variation of the osteomeatal complex region. Its incidence has been reported between 13% and 53% [1, 2]. The development mechanism pneumatization of the nasal turbinates is still unknown. Several theories have been proposed for its occurrence: some of them are expansion of sinus pneumatization into the turbinate during intrauterine period, fusion abnormality during intrauterine development, and conchal bone microfractures during late puberty causing nasal mucosal invagination expanding to bullosa cavity. Also, two different theories have been suggested on this issue by Stammberger [3]. According to the first theory, after the formation of septum deviation, the air flow pattern of nasal cavity and on the opposite side of the space provokes the development of CB. According to another theory, the CB and septal deviation are two different anomalies. It has been shown that anterior and posterior ethmoidal air cells are liable for pneumatization of CB roughly in 55% and 45% of the cases, respectively [1, 2].

Although the majority is asymptomatic, due to its negative effect on paranasal sinus ventilation and mucociliary cleaning of the middle meatus, CB can cause the development of maxillary or ethmoidal sinusitis. The severity of symptoms resulting from CB is closely associated with the degree of pneumatization.

The difference between a hypertrophied turbinate and its pneumatization can be only distinguished by paranasal sinus CT. In our patient, the anterior rhinoscopic and endoscopic views of the nasal cavity were not distinctive for pneumatization of the turbinates, but paranasal sinus CT imaging allowed us to diagnose the pathology. To our knowledge, there have been two reports about a case with CB in all turbinates [4, 5]. We present the third case of a patient with bilateral pneumatization in three intranasal turbinates.

The treatment of CB is endoscopic partial middle turbinate resection. With the widespread use of imaging techniques such as CT have provided us with detailed information of nasal cavity and paranasal sinuses. This helps the surgeons be aware of the anatomic variations regarding the osteomeatal complex region. Surgical resection of CB during endoscopic sinus surgery requires careful protection of medial lamella and resection of only lateral half of turbinate. The extent of turbinate pneumatization is evaluated on paranasal sinus CT scans and this allows the surgeon to anticipate points of safe entry into the lumen of CB. Careful evaluation of paranasal sinus CT scans before surgery is important in these cases.

## 4. Conclusion

Bilateral triple CB is very rare anatomical variation. The presence of such anatomical variations regarding intranasal nasal turbinates can lead to complications during endoscopic sinus surgery. The anatomical variations of the intranasal turbinates can be determined with paranasal sinus CT. Therefore, it is especially important that otolaryngologists and radiologists are well aware of such anatomical variations of osteomeatal complex region when evaluating patients with sinonasal symptoms.
